# Hand, Foot, and Mouth Disease Caused by Coxsackievirus A6, Thailand, 2012

**DOI:** 10.3201/eid1904.121666

**Published:** 2013-04

**Authors:** Jiratchaya Puenpa, Thaweesak Chieochansin, Piyada Linsuwanon, Sumeth Korkong, Siwanat Thongkomplew, Preyaporn Vichaiwattana, Apiradee Theamboonlers, Yong Poovorawan

**Affiliations:** Chulalongkorn University, Bangkok, Thailand

**Keywords:** hand, foot, and mouth disease, herpangina, enterovirus 71, coxsackievirus A6, coxsackievirus A16, Thailand, viruses

## Abstract

In Thailand, hand, foot, and mouth disease (HFMD) is usually caused by enterovirus 71 or coxsackievirus A16. To determine the cause of a large outbreak of HFMD in Thailand during June–August 2012, we examined patient specimens. Coxsackievirus A6 was the causative agent. To improve prevention and control, causes of HFMD should be monitored.

## Abstract 2

Coxsackievirus A6, Thailand

Coxsackievirus A6 (CAV6) is 1 of 10 genotypes within the family *Picornaviridae*, genus *Enterovirus*, species *Human enterovirus A*. Other genotypes include coxsackievirus A16 (CAV16) and enterovirus 71 (EV71). Although CAV6 is commonly associated with hand, foot, and mouth disease (HFMD) and herpangina (*1*,*2*), it has not been of concern until the recent global outbreaks of HFMD (*3*–*6*).

In Thailand, the viruses predominately associated with HFMD have been EV71 and CAV16 (*7*,*8*); to our knowledge, CAV6 has not been implicated. In 2012, extensive outbreaks of HFMD occurred in Thailand. To determine the pattern, causative agents, and clinical manifestations of HFMD in this 2012 outbreak, we analyzed specimens from patients. This study was approved by the institutional review board of the Faculty of Medicine, Chulalongkorn University; the requirement for written informed consent was waived because the samples were analyzed anonymously.

## The Study

In Thailand, HFMD usually occurs during the rainy season (June–August); average incidence during 2007–2011 was 20.2 cases per 100,000 population (*9*,*10*). In 2012, an extensive outbreak of HFMD occurred; the incidence rate was 3-fold higher than the average incidence rate of 58.15 cases per 100,000 population or >36.000 cases; the 2012 outbreak included 2 fatal cases of EV71 encephalitis (*11*). In this outbreak, 2 clinical patterns were observed, and 2 case definitions were applied. Suspected HFMD cases were defined as painful blisters in the oropharynx and blisters on the palms, soles, knees, elbows, and/or buttocks. Suspected herpangina cases were defined as painful blisters in the mouth only, predominantly on the soft palate. Suspected HFMD and herpangina cases were virologically confirmed if samples were positive for viral RNA by nested PCR. 

**Figure 1:**
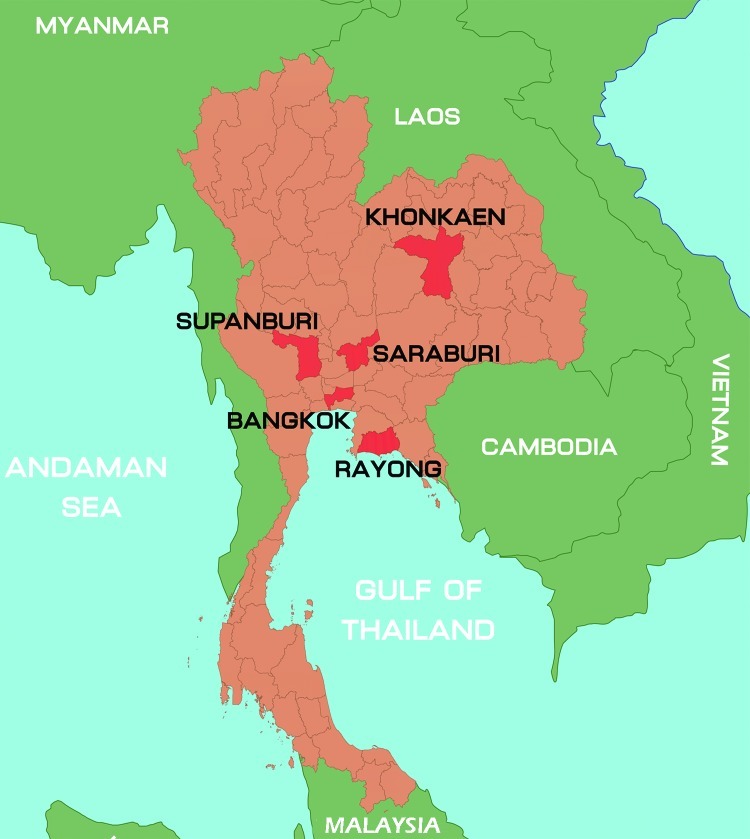
Location of sample collection sites during outbreak of hand, foot, and mouth disease, Thailand, January–October 2012.

During January–October 2012, a total of 847 samples were collected from 825 patients with suspected cases. Among those 825 patients, the diagnosis was HFMD for 672 (81.4%) and herpangina for 153 (18.6%). Patients’ ages ranged from 1 month to 38 years. The samples were collected from hospitalized patients and outpatients who had a clinical diagnosis of HFMD or herpangina and who came from different parts of Thailand: Bangkok, 566 cases; Khonkaen, 252 cases; Suphanburi, 4 cases; and Saraburi, Rayong, and Chantaburi, 1 case each (Figure 1). Of the 847 samples, 695 were rectal swabs, 73 fecal, 39 throat swabs, 20 serum, 9 vesicle fluid, 7 nasal swabs, 3 cerebrospinal fluid, and 1 saliva. All samples, other than stool samples, were collected in virus transport media modified according to recommendations by the World Health Organization (*12*). Fecal samples were diluted 1:10 with phosphate-buffered saline and centrifuged, and the supernatant was collected for testing. Viral RNA was extracted from 200-μL samples by using the Viral Nucleic Acid Extraction Kit (RBC Bioscience, Taipei, Taiwan) according to the manufacturer’s instructions. cDNA was synthesized by using the ImProm-II Reverse Transcription System (Promega, Madison, WI, USA) with random hexamers as primers according to the manufacturer’s recommendation (First BASE Laboratories, Selangor Darul Ehsan, Malaysia). 

To identify enteroviruses, we performed 3 separate PCRs. The first PCR, which could detect most enteroviruses, was used to screen for panenterovirus. The 5′ untranslated region of the viruses was amplified by nested PCR as described (*13*). The second PCR was selective for EV71 and CAV16; the primers and reaction conditions were identical to those used in a previous study (*7*). The third PCR, for CAV6 detection, used primers designed to amplify the viral protein (VP) 1 gene by seminested PCR with CU-EVF2632 (5′-TGTGTGATGAATCGAAACGGGGT-3′) and CU-EVR3288 (5′-TGCAGTGTTAGTTATTGT TTGGCT-3′) as first-round primers and CU-EVR3053 (5′-GGGTAACCATCATAAAACCACTG-3′) as a reverse primer for the second round. The expected 420-bp PCR product was examined under UV light after being resolved in 2% agarose gel electrophoresis and subsequently stained with ethidium bromide.

**Figure 2:**
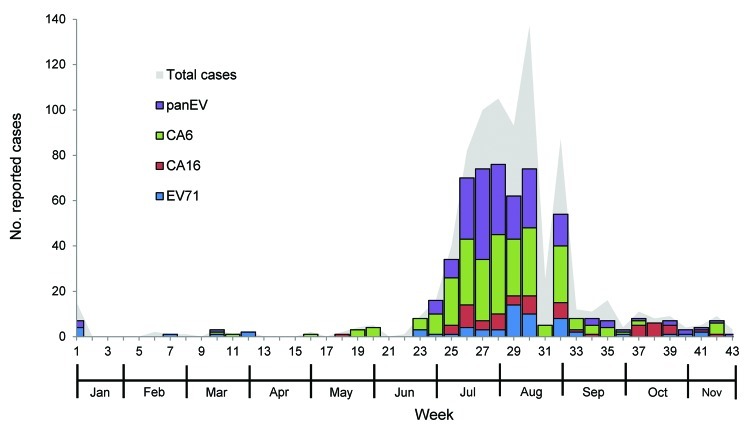
Weekly number of reported suspected cases of hand, foot, and mouth disease and herpangina during outbreak, Thailand, 2012. EV, enterovirus; CA6, coxsackievirus 6; CA16, coxsackievirus 16; EV71, enterovirus 71.

Most samples were collected during the rainy season, from the end of June through early August 2012 (weeks 25–32), which accounted for 83.1% of all reported cases. Altogether, enterovirus results were positive for 459 (68.3%) HFMD and 101 (66.0%) herpangina patients (Figure 2),

Of note, 93.1% of patients were <5 years of age. A high proportion of cases was found among children 1, 2, and 3 years of age and accounted for 68.4% of HFMD cases and 64.2% of herpangina cases (Technical Appendix Figure 1).

Of the 672 HFMD cases, 221 (32.9%) were caused by CAV6, 62 (9.2%) by EV71, 62 (9.2%) by CAV16, and 114 (17.0%) by untyped enteroviruses. Of the herpangina cases, 13.7% were caused by CAV6 and 1.3% by CAV16. Moreover, samples from 51.0% of patients with herpangina were positive for an untyped enterovirus (Table).

**Table:** Causative agents identified during hand, foot, and mouth disease outbreak, Thailand, 2012

Virus	No. (%) cases	
	Hand, foot, and mouth disease	Herpangina
Coxsackievirus A6	221 (32.9)	21 (13.7)
Coxsackievirus A16	62 (9.2)	2 (1.3)
Enterovirus A71	62 (9.2)	0
Panenterovirus only	114 (17.0)	78 (51.0)
None detected	213 (31.7)	52 (34.0)
Total	672	153

Generally, the clinical manifestations of HFMD were fever; drooling, and refusal to eat (among young children); painful lesions in the mouth, especially on the soft palate (Technical Appendix Figure 2, panel A); and vesicular rashes on the palms and feet (Technical Appendix Figure 2, panels B, C). For patients affected by this outbreak, physicians from reporting sites reported anecdotally that they observed more severe skin manifestations than usual, especially on the buttocks and perianal area (Technical Appendix Figure 2, panel D), knees, and elbows. Two cases with neurologic involvement (convulsion, altered consciousness) were caused by EV71 and were treated with intravenous immunoglobulin. No patients died.

Direct sequencing was performed on the VP1 region of 143 randomly selected CAV6-positive samples. The sequences were submitted to GenBank under accession nos. JX556422–JX556564.

The VP1 nucleotide sequences of CAV6 were aligned with the reference sequences by using ClustalW in the BioEdit program version 7.0.9.0 (www.mbio.ncsu.edu/BioEdit/bioedit.html). A phylogenetic tree was constructed with MEGA software, version 5.0, by applying the maximum-likelihood method and using the Kimura 2-parameter model, in which 1,000 replications were selected for bootstrapping (*14*) (Technical Appendix Figure 3). The sequences of EV71 strain BrCr (accession no. U22521) and CAV16 strain G10 (accession no. U05876) were used as outgroups in the phylogenetic analysis.

The relationship between the CAV6 characterized in this study and the prototype strain (Gdula) was investigated by phylogenetic analysis of partial VP1 sequences. All CAV6 clustered in the same lineage and with the reference strain CAV6 (Gdula); nucleotide homologies among these strains were 81.4%–84.7%.

## Conclusions

Although the positive samples collected during January–October 2012 were mostly from patients in Bangkok and Khonkaen, they partially represented the HFMD and herpangina cases in Thailand’s 30,000-case outbreak. Virus prevalence in Thailand was highest in HFMD and herpangina patients 1–3 years of age (Technical Appendix Figure 1). For this seasonal outbreak, the most common causative agent was CAV6. All CAV6 strains shared an isolated cluster and had high similarity, as shown in the phylogenetic analysis of VP1 region. Although CAV6 has been a predominant emerging pathogen since 2012, no patients infected with CAV6 died. According to the study conducted during 2008–2011 EV71 and CAV16 were the main pathogens contributing to the disease (*7*). However, we found a different main pathogen: CAV6. For prevention and control of future outbreaks, the causes of HFMD should be monitored.

Technical Appendix Age distribution of patients with reported cases of hand, foot, and mouth disease and herpangina, Thailand, January–October 2012, clinical manifestations in children with coxsackievirus A6 infection, and phylogenetic analysis of coxsackievirus A6.
