# Supplementary material for: Hand, Foot, and Mouth Disease Caused by Coxsackievirus A6, Thailand, 2012
Source: Emerg Infect Dis. 2013 Apr;19(4):641–3. doi: 10.3201/eid1904.121666 (PMC3647428; doi:10.3201/eid1904.121666)
Supplement: Technical Appendix — Age distribution of patients with reported cases of hand, foot, and mouth disease and herpangina, Thailand, January–October 2012, clinical manifestations in children with coxsackievirus A6 infection, and phylogenetic analysis of coxsackievirus A6. [file 12-1666-Techapp-s1.pdf]

Article DOI: http://dx.doi.org/10.3201/eid1904.121666

Outbreak of Hand, Foot, and Mouth Disease Caused by Coxsackievirus A6, Thailand, 2012

Technical Appendix

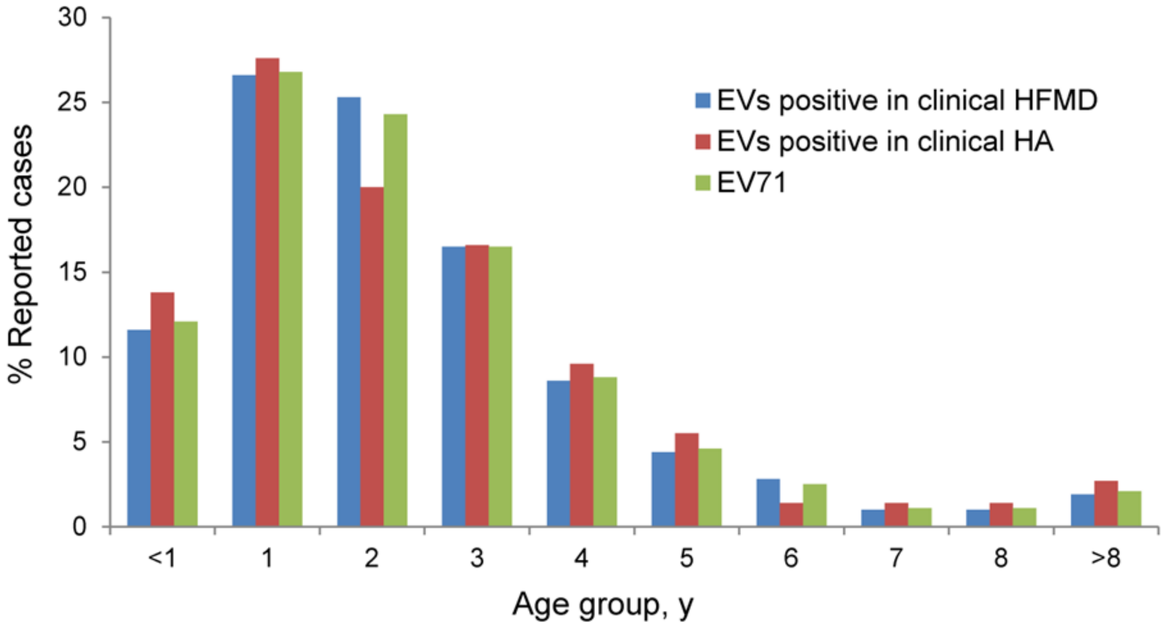

Figure 1. Age distribution of patients with reported cases of hand, foot, and mouth disease (HFMD) and herpangina (HA), Thailand, 2012. EV, enterovirus.

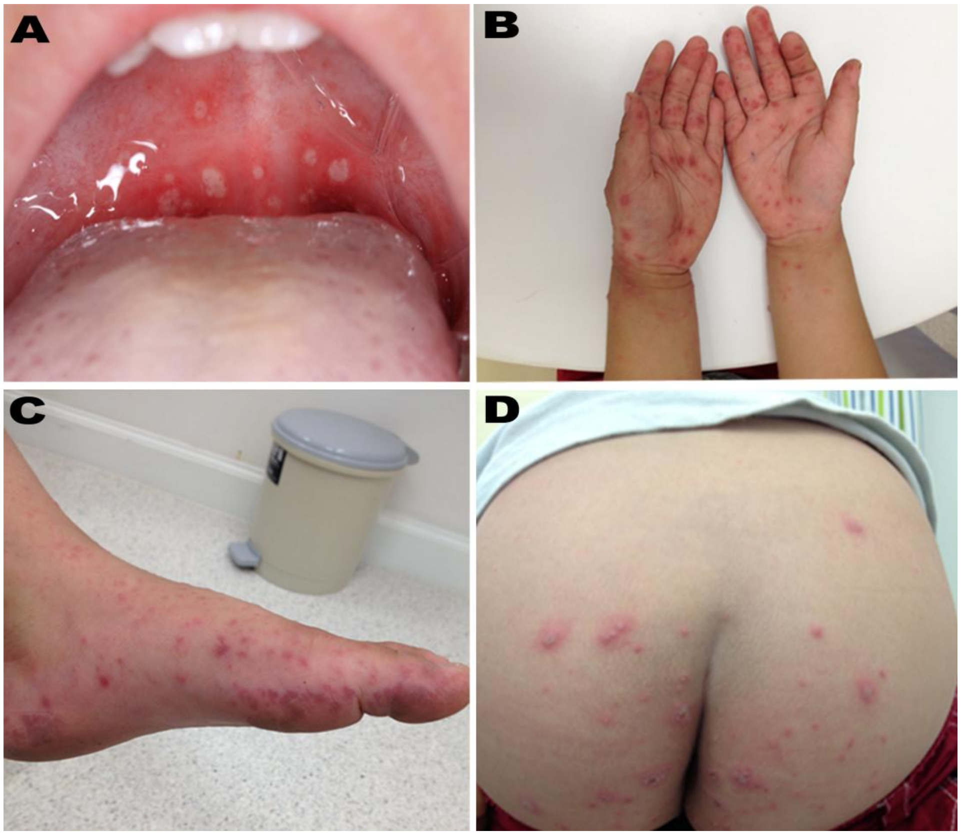

Technical Appendix Figure 2. Clinical manifestations in children with coxsackievirus A6 infection during outbreak of hand, foot, and mouth disease and herpangina, Thailand, 2012. A) Lesions on the soft palate of the mouth; B) vesicular rash on the hands; C) vesicular rash on the foot; D) lesions on the buttocks and perianal area.

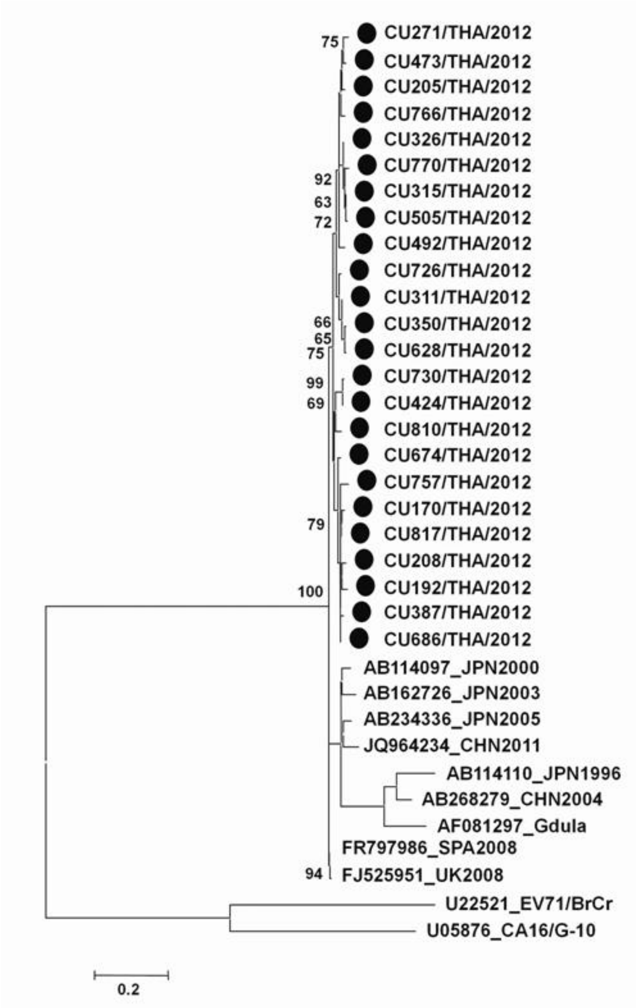

Technical Appendix Figure 3. Phylogenetic analysis of coxsackievirus A6 from affected children during outbreak of hand, foot, and mouth disease and herpangina, Thailand, 2012. Tree is based on partial viral capsid protein 1 nucleotide sequences (nt 2646–3042). Scale bar indicates branch distances; solid circles indicate strains from the outbreak.
